# Dietary Component-Induced Inflammation and Its Amelioration by Prebiotics, Probiotics, and Synbiotics

**DOI:** 10.3389/fnut.2022.931458

**Published:** 2022-07-22

**Authors:** Muhammad Bilal, Shoaib Ashraf, Xin Zhao

**Affiliations:** Department of Animal Science, McGill University, Sainte-Anne-de-Bellevue, QC, Canada

**Keywords:** inflammation, probiotics, prebiotics, dysbiosis, western diet, synbiotics, undernutrition, microbiota

## Abstract

A balanced diet with many dietary components maintains immune homeostasis directly by interacting with innate and adaptive immune components or indirectly through gut microbiota and their metabolites. Dietary components may inhibit pro-inflammatory mediators and promote anti-inflammatory functions or vice versa. Western diets with imbalanced dietary components skew the immune balance toward pro-inflammation and induce intestinal inflammation, consequently leading to many intestinal and systemic inflammatory diseases like ulcerative colitis, Crohn’s disease, irritable bowel syndrome, cardiovascular problems, obesity, and diabetes. The dietary component-induced inflammation is usually chronic in nature and frequently caused or accompanied by alterations in gut microbiota. Therefore, microbiome-targeted therapies such as probiotics, prebiotics and synbiotics hold great potentials to amend immune dysregulation and gut dysbiosis, preventing and treating intestinal and systemic inflammatory diseases. Probiotics, prebiotics and synbioitcs are progressively being added to foods and beverages, with claims of health benefits. However, the underlining mechanisms of these interventions for preventing and treating dietary component-induced inflammation are still not very clear. In addition, possibly ineffective or negative consequences of some probiotics, prebiotics and synbiotics call for stringent testing and regulation. Here, we will first briefly review inflammation, in terms of its types and the relationship between different dietary components and immune responses. Then, we focus on current knowledge about the direct and indirect effects of probiotics, prebiotics and synbiotics on intestinal and systemic inflammation. Understanding how probiotics, prebiotics and synbiotics modulate the immune system and gut microbiota will improve our strategies for preventing and treating dietary component-induced intestinal inflammation and inflammatory diseases.

## Introduction

To improve gut health and counter dietary component-induced inflammation, microbiome-targeted therapies hold enormous potentials to ameliorate gut dysbiosis and associated immune dysregulation. Currently, one type of microbiome-targeted therapies, fecal microbiota transplantation, is widely used in clinical practice to treat recurring *Clostridioides difficile* infections. Other types of microbiome-targeted therapies include usage of probiotics and prebiotics, which are progressively being added to foods and beverages, with claims of health benefits. However, the underlining mechanisms for these interventions to impact host health such as dietary component-induced inflammatory conditions remain elusive.

Dietary components have the potential to generate both pro- and anti-inflammatory signals that can modulate gut immune responses. Different dietary nutrients interact directly with innate and adaptive immune components or indirectly through mediation of gut microbiota and their metabolites to impact immune responses (1, 2). Gut microbiota is sensitive to dietary constituents and alterations in diet can cause large shifts in microbial composition (3, 4). Based on composition of microbial communities, gut microbiota passes on both pro- and anti-inflammatory stimuli to the immune system and thus act as a facilitator through which food components employ their anti-inflammatory and pro-inflammatory effects. For instance, animal trials have showed that foods with high concentrations of saturated fats (5) and sugar (6) induce inflammatory and autoimmune conditions through microbial processes including stimulation of T-helper 17 (TH17) cells. In opposite, tryptophan metabolites suppress inflammation through activation of the aryl hydrocarbon receptor (AhR) in lymphoid tissues and generation of regulatory T (Treg) cells (7, 8). Similarly, high intake of dietary fiber promotes microbial population producing short chain fatty acids (SCFA) that activate Treg cells through G protein coupled receptor 43 (GPR43) receptors to suppress inflammation (9).

Unlike Mediterranean diets which are plant based and have olive oil as the main source of added fat, Western diets (defined as high content of saturated fats and sucrose and low content of fiber) are usually deficient in advantageous micronutrients (vitamins) and trace-elements (zinc, phosphorus, calcium, magnesium, or potassium) and abundant in detrimental elements such as sugar, unhealthy fats, salt, and refined grains (10). Mediterranean diets mainly include whole grains, vegetables, legumes, fruits, nuts, seeds, herbs, and spices. Olive oil is the main source of added fat. Fish, seafood, dairy, and poultry are included in moderation. Red meat and sweets are eaten only occasionally. On the other hand, Western diets mostly contain high amounts of processed foods, red meat, high-fat dairy products, high-sugar foods, and pre-packaged foods. This dietary configuration in Western diets promotes pro-inflammatory alterations in the gut microbial diversity (decrease in microbial diversity) and composition (such as increase in the Firmicutes to Bacteroidetes ratio), which lead to compromised gut integrity (increase in epithelial barrier permeability) and activate pattern-recognition receptors (PRR) like toll-like receptor (TLR) 4 that consequently activate pro-inflammatory mediators such as cyclooxygenase 2 (COX2), tumor necrosis factor-α (TNF-α), interleukin-1β (IL-1β), interleukin-6 (IL-6), interleukin-8 (IL-8), interleukin-12 (IL-12), and interferon-γ (IFN-γ) (2). Such dietary induced inflammation associated with impaired immune functions and gut dysbiosis may consequently lead to an “inflammatory disorder” such as Crohn’s disease (CD), ulcerative colitis (UC), and irritable bowel syndrome (IBS) (11, 12). For example, patients with IBS had more Firmicutes and less Bacteroidetes than controls and a decrease in *Faecalibacterium prausnitzii* accompanied by an increase in the abundance of *Streptococcus* species was the main characteristic of the gut microbiota of participants with IBS symptoms (13). In addition, the gut microbiota also impacts the systemic immunity and is involved in many systemic immune-mediated inflammatory diseases (IMIDs), ranging from diabetes to arthritis and systemic lupus erythematosus (14). Emerging evidence indicates that metabolic disorders such as obesity, type 2 diabetes mellitus (T2DM) and non-alcoholic fatty liver disease (NAFLD) are characterized by alterations in the intestinal microbiota composition and its metabolites, which translocate from the gut across a disrupted intestinal barrier to affect various metabolic organs, such as the liver and adipose tissue, thereby contributing to low-grade metabolic inflammation (15).

Altering the gut microbial population by leveraging probiotics, prebiotics and synbiotics is becoming effective and popular modalities for disease prevention and treatment. In recent years, probiotics, prebiotics and synbiotics are increasingly being incorporated into a wide range of foods, beverages, and topical products (16). However, the impact of microbiome-targeted interventions such as probiotics, prebiotics and synbiotics on dietary component-induced inflammation and metabolic diseases is still scarce and further investigations are required both in healthy as well as in disease conditions. This is further complicated by the fact that most functional experiments have been carried out with individual dietary components. While these studies have enhanced our understanding on pro- and anti-inflammatory abilities of single compounds, the knowledge related with the impact of whole food and dietary patterns is less clear. Thus, it is timely to review their potential roles in relieving inflammation.

## Inflammation

**FIGURE 1:**
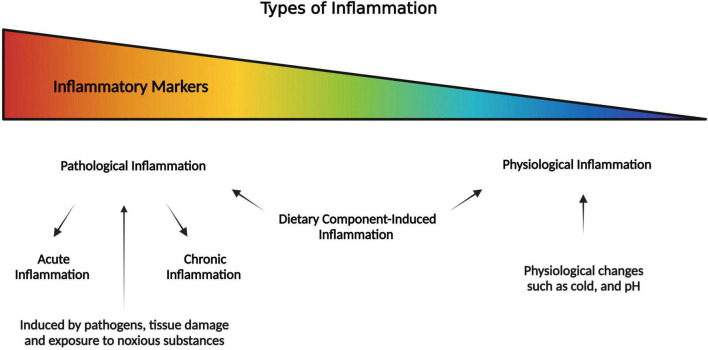
Pictorial representation of the spectrum of inflammation where it is pathological inflammation at one extreme, and physiological inflammation at the other extreme (as part of normal homeostasis). In the middle of the spectrum is the dietary component-induced inflammation which is a special case that may lead to pathological or physiological inflammation.

Inflammation is a conundrum that defies a simple definition and can be conceptualized on a “spectrum.” Thus, inflammation can be defined as a biological response of the body to different perturbations (injury or infection) or to maintain homeostasis (17). Inflammation can be classified in many ways such as being acute or chronic based on the length of inflammation, and physiological or pathological based on presence or absence of pathological agents. The hallmarks of the most familiar type of inflammation include cardinal signs such as redness, swelling, heat, and pain. Inflammation is prompted by innate immune responses that identify infection, host damage, and danger signaling molecules and stimulate a regulated system of immunological and physiological events to sustain homeostasis as well as restore functionality (18). However, an increasing number of evidence suggest that cells and mediators that are essential for inflammation are equally important in homeostatic functions such as metabolism, tissue remodeling, and interorgan crosstalk (17). Hence, inflammation can be contextualized in the terms of a response, a process, or a state of the system. Each of these phenomena emphasize different aspects of inflammation. For example, (1) as a response to perturbations, (2) to eliminate source of perturbation, and (3) alter the state of system which could be either protective or pathological. As shown in Figure 1, one extreme of the spectrum of inflammation is pathological inflammation (pathogen induced) that include acute inflammation (if tissue can quickly recover) and chronic (if the pathology prolongs for a long period). At the other end of the spectrum is physiological inflammation (as a part of normal homeostasis), in responses to non-pathogens such as cold or fasting. The dietary component-induced inflammation (also non-pathogenic) exists in between the spectrum of pathologic and physiologic inflammation.

### Pathological Inflammation

Pathological inflammation can be defined as a diseased process that results from abnormal or disrupted interactions between immune and non-immune components. Pathological inflammation can be a self-limiting or never-ending condition with a complete or incomplete resolution, respectively. At the intestinal surface pathological inflammation leads to chronic intestinal inflammation, which is typically observed in patients with CD or UC (19). Following an acute microbial infection or tissue injury, the host defense and tolerance to commensals is perturbed and physiological inflammation changed to a true inflammatory response or pathological inflammation (20). It is characterized by an acute microvascular response, edema, and stimulation of monocytes and neutrophils (21). This type of inflammation intends to eradicate the source of perturbation, repair the damaged structures and supplement tissue structures with alterations that optimize defenses. For instance, intestinal epithelial lining goes through inflammatory transformation and increase the number of mucus-producing goblet cells and alters mucus structure and composition (22). Pathological inflammation can be further divided into acute inflammation and chronic inflammation, based on the length of inflammation.

#### Acute Inflammation

Acute inflammation is an immediate adaptive response due to noxious stimuli and has limited specificity. If the inflammatory response is controlled, it can be beneficial to the body and could provide protection against many infectious organisms. Contrary to this, if it is uncontrolled, it can be detrimental such as in the case of septic shock. Acute inflammation follows a sequence of events and involves inducers, sensors, mediators, and effectors (23, 24). Inducers can be infectious agents [for example, pathogen associate molecular patterns (PAMPs) present on all microorganisms or virulence factors specific to pathogens] or non-infectious stimuli (for example, allergens, toxicants, physical injury, or signals released by cells/tissues that are malfunctioned or dead). The inducers then activate sensors which will stimulate mediators. Examples of sensors include TLR4, immunoglobulin-E (IgE), Nacht Domain-, Leucine-Rich Repeat-, and PYD-Containing Protein 3 (NALP3), Hageman factor and purinoceptors that are expressed on specialized cells. Mediators are specialized entities (for example, TNF-α, IL-6, bradykinin, IL-1β, arachidonic acid, mast cells, and complement) that are activated by inducers through sensors and can modulate inflammation by activating the effectors, which are tissues or cells (17, 25). These players can act in parallel on multiple inflammatory pathways depending upon the stimuli with a goal to restore homeostasis regardless of the cause. The features of acute inflammation include vasodilation, increase in blood flow, capillary permeability, and migration/sequestration of neutrophils to the infected site through diapedesis (26).

#### Chronic Inflammation

Chronic inflammation is often referred to as slow or long-term inflammation and ranges from months to years. The extent and effects of chronic inflammation depend upon the cause of the injury and the ability of the body to respond to the stimulus in terms of repairing and overcoming the assault (26). Some examples of the etiologies of chronic inflammation include: (1) persistence of infectious organisms in the body due to evasion of the host defenses, for example, bacteria, parasites, and fungi, (2) low level exposure to foreign materials that cannot be eliminated by the body such as dust, (3) autoimmune disorders that attack the healthy tissues of the body such as rheumatoid arthritis, and systemic lupous erythematosus, (4) recurrent episodes of acute inflammation, (5) inducers of continuous oxidative stress and mitochondrial dysfunction, for example, urates, and increased production of free radical, and (6) excessive nutrient intake or the inability of the body to metabolize nutrients. Chronic inflammation follows the same pattern as acute inflammation except for the composition of white blood cells, for instance, the plasma cells, macrophages, and lymphocytes replace the neutrophils. These cells reach the site of injury and start producing cytokines, and enzymes leading to fibrosis and granuloma formation. One special cause/type of chronic inflammation is due to metabolic disorders, nutrition or an impaired gut-microbiome referred to as metabolic inflammation and will be discussed in detail below.

### Physiological Inflammation

Physiological intestinal inflammation is proposed as a normal response that prevents gut injury through its ability to flawlessly adapt to multiple proinflammatory challenges and is therefore essential to health (19). Physiological inflammation occurs in the absence of any infection, tissue damage or exposure to noxious substances. As such, physiological inflammation can also occur in response to environmental stimuli such as cold and fasting that lead to impaired homeostasis. Some examples of physiological inflammation include control of metabolic homeostasis, regulation of thermogenesis and in the case of flight or fright responses (17). Hence, physiological inflammation is a physiological process of the body that acts as a defense mechanism to maintain homeostasis. Physiological inflammation at the intestinal level is maintained by gut microbiota through direct or indirect stimulation from the non-pathogenic commensals (27). Here, to maintain physiological balancing, the commensals stimulate production of physiological concentrations of proinflammatory cytokines such as IL-1β, IL-6, lymphotoxin, and TNF-α to maintain mucosal homeostasis (28, 29). Physiological inflammation that results in intestinal homeostasis are the result of normal interactions between the immune and non-immune cells at the normal intestinal mucosal surface. During physiological inflammation, the intestinal immune system and gut microbiome live in a mutually beneficial homeostasis sharing a bi-directional relationship, with both components affecting each other’s functionality, composition, and development (30). Under these conditions, the intestinal immune system performs defense against the attack of pathogenic organisms and maintain a state of tolerance to the commensal microbes in the gut (31). In addition to the role of gut microbiota, the metabolites (such as SCFAs and amino acids) derived from microbiota have also emerged as important regulators of homeostasis and inflammation. SCFA are formed as metabolites from the dietary fibers taken up by intestinal microbiota. Acetate, propionate, and butyrate are the most abundant SCFAs. SCFAs can maintain intestinal homeostasis through controlling inflammation via activation of the NLR Family Pyrin Domain Containing 2 (NLRP2) inflammasome by G-protein-coupled receptors (GPCRs). Alternatively, SCFAs can be taken up by the cells and regulate inflammation through intracellular receptors such as peroxisome proliferator-activated receptor γ (PPARγ). Among the SCFAs, butyrate is particularly known to promote epithelial barrier integrity through tight junctions (TJ), and mucin proteins. Similarly, amino acids like tryptophan and phenylalanine play important roles for the host. Tryptophan plays a key role in host immunity through activation of aryl hydrocarbon receptor (AhR) and kynurenine pathway through indoleamine 2,3-dioxygenase (IDO) (32). Dietary component induced inflammation can be viewed as a special type of physiological chronic low-grade inflammation, which may lead to metabolic disorders such as obesity, atherosclerosis, (T2DM) and NAFLD. Like most chronic inflammations this type of inflammation is also characterized by low- level local or systemic inflammatory responses (15). It is caused by excessive nutrient intake, which promotes metabolic dysfunction through activation of the same signal transduction mediators and pathways as immune responses to infections (33). The argument in support of the sterile hypothesis is that excess dietary lipids resulting in lipotoxicity (in liver and adipose tissue) were reported as the cause of this phenotype (metabolic inflammation), in the absence of an infectious agent (34–36). However, contrary to this the evidence that supports the non-sterile hypothesis comes from the involvement of the breech at the gastrointestinal tract interface which harbors many bacteria. Healthy individuals have an organized intestinal barrier which prevents dissemination of bacteria and have a diverse composition of gut microbiota. Unlike healthy individuals, people with obesity or other metabolic disorders (T2DM, and NAFLD, etc.) exhibit excessive alterations in the gut microbiota (a term called dysbiosis) along with having an impaired intestinal barrier (15). To this end, dysbiosis and a damaged intestinal barrier may result in an altered pool of commensals that might translocate into the circulation and elicit low grade inflammation which is often detrimental to the host. Furthermore, high fat diet has also been associated with gut dysbiosis, altered functional and compositional changes in the microbiota, along with intestinal barrier disruption that leads to translocation of lipopolysaccharides (LPS) as well as endotoxemia (15). The innate immune cells like macrophages recognize harmful stimuli like LPS and lipids, saturated fatty acids, with PRR like TLR-4 and activate inflammatory pathways like the nod-like receptor containing a pyrin domain (NLRP3) inflammasome. The activation process of inflammasome happens through two-step mechanism. The first priming step entails activation of the TLR and signaling molecules like MyD88 through ligands that further activate the transcription factor nuclear factor kappa B (NF-kB), transcription factor that control most of the pro-inflammatory mediators, which results in the transcription of inactive forms of inflammatory cytokine, IL-1β and IL-18. In the second step inflammasome is activated in response to cell stress signals and activates the protease caspase-1, which cleaves pro-IL-1β and pro-IL-18 into active secreted forms and proceed inflammation (37). Following inflammation, the resolution of inflammation is an active metabolic inflammatory process involving removal of apoptotic neutrophils and clearance of phagocytes (38). This process is mediated through different mediators like resolvins and protectins, and any disruption in their biosynthesis may lead to failure in resolution and consequently chronic inflammation (39). In addition, the adaptive immune cells also reported to show pro-inflammatory configuration in metabolic inflammation. The population of T helper 17 (Th17) and CD8+ T cells has been increased while IL-10 producing anti-inflammatory Treg cells population has been declined (15). The Treg cells play a pivotal role in the regulation of responses of innate immune cells and thus influence the inflammatory processes (40). While dietary component-induced inflammation can be initially considered as a special case of the physiological inflammation, chronic dietary component-induced inflammation can lead to pathological inflammation and subsequently metabolic diseases.

## Pro-And Anti-Inflammatory Microbiota and Their Interactions With Host Immune System

The intestinal tract holds a diverse community of microbes that are evolved with the host immune system and have profound interactions with each other. Microbiota is needed for gut immune development while immune responses regulate the composition and structure of the gut microbiota (41). Thus, changes in the microbiota may have a consequent effect on the host. Gut microbiota includes many different types of bacteria, scattered in a high number of taxa with complex ecological relationships among them and with the host. The abundance of certain taxa has been linked with pro-inflammatory or anti-inflammatory consequences. For instance, decreases in Firmicutes and Bacteroidetes and increases in Enterobacteriaceae (e.g., *Escherichia coli*) has been reported in patients with inflammatory conditions like inflammatory bowel disease (IBD) (42). Similarly, strains of adherent and invasive *E. coli* (43) and *Enterococcus faecalis* (44) are commonly identified in the mucosa of CD patients. Additionally, certain pro-inflammatory bacterial strains, such as *Ruminococcus gnavus* or *Bacteroides* species, might dominate in metabolic inflammatory conditions like obesity (45). In contrast, other commensal microbial taxa in the gut presents anti-inflammatory roles and their lower abundance in the gut promotes inflammation. For example, reduction in *F. prausnitzii* (46), *Lactobacillus GG* (47), and *Bifidobacterium* species (48) have been associated with inflammatory conditions. Similarly, *clostridia* strains falling within clusters IV, XIVa, and XVIII have been associated with expansion and differentiation of anti-inflammatory Treg cells of the host and helped in attenuation of inflammatory conditions of colitis and allergic diarrhea (49). Keeping in view the presence of diverse microbial communities with diverse pro- and anti-inflammatory capabilities to interact with the host immune system, it is plausible to think that they may communicate a mix of pro- and anti-inflammatory signals and maintain immune homeostasis. It is important to understand that dietary interventions are promoting which segments of microbiota, favorable or harmful, and whether they facilitate inflammatory or anti-inflammatory immune responses. It will also be important to realize that skewing immune homeostasis too much toward anti-inflammatory arm would interfere with routine immune responses to pathogens, cancer cells and vaccines.

**FIGURE 2:**
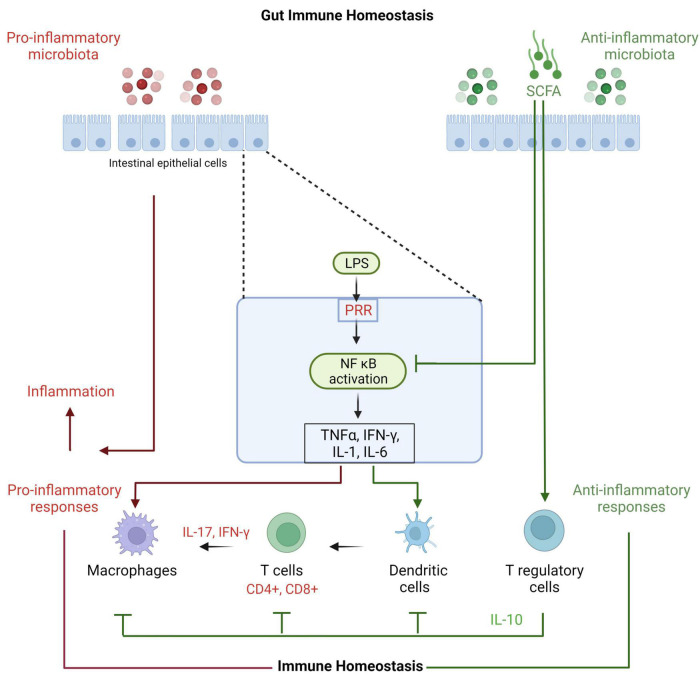
Effects of microbiota on gut immune homeostasis. Gut microbiota and their metabolites interact with gut epithelial cells through PRR such as membranous Toll-like receptors and cytoplasmic NOD-like receptors. The subsequent inflammatory mediators activate other immune components. The gut immune system protects the epithelial lining and responds to incursions with different immune cells including dendritic cells, macrophages, CD4 and CD8 T cells and their cytokines like gamma interferon and IL-17 to proceed inflammation. IL-17 is important as it stimulates tight junction and mucin proteins and improves gut integrity and function. Longer and exaggerated inflammation is not helpful and counterproductive. Thus, T reg cells with the help of its key IL-10 cytokine control these cells and maintains immune homeostasis.

The gut contains a plethora of microorganisms that are a source of PAMPs and metabolites (Figure 2). PAMPs interact with the host by provoking responses through PRR such as TLR, NOD-like receptors and RIG-I-like receptors (50). Intense functional and compositional alterations in the gut microbiota may lead to dysbiosis (39), which is commonly associated with a bloom of commensals that may become detrimental (51). The dysbiosis may promote pro-inflammatory microbiota as well as their interactions with the host, which is followed by imbalanced immune response with high expression of innate pro-inflammatory cytokines, including TNF-α, IL-6, and IL-1β and T helper (Th)1 and Th17 (IL-17a) cells activation that result in local inflammation (2). An impaired and defective intestinal barrier result in translocation of microbiota or its components (like lipopolysaccharides) into the circulation and consequently initiation of systemic low- grade inflammation (15). On the other hand, microbial communities with anti-inflammatory capabilities, like lactic acid bacteria and Bifidobacteria, are known to produce factors that inhibit inflammation through downregulating IL-8 secretion, NF-κB dependent gene expression and macrophage-attracting chemokines (52). Bifidobacteria and lactic acid bacteria are also involved in direct downregulation of T effector-mediated inflammatory responses and upregulation of anti-inflammatory Treg cells expression in mice (53). Several studies suggest that microbial-derived SCFA may be adding via G-protein-coupled receptor and epigenetic mechanisms (54, 55). Intestinal SCFAs may directly increase the abundance of T reg cells in the gut (56) or may inhibit the transcription factor NF-κB, leading to decreased secretion of pro-inflammatory cytokines and inhibition of inflammation (57). In addition, microbial-derived butyrate inhibits histone deacetylases 6 and 9, which increases acetylation in the fork head box P3 (FOXP3) promoter gene and higher Treg cells proliferation (55). Clearly the gut microbiota presents diverse microbiota with both pro- and anti-inflammatory interactions with the host immune system, and a balanced gut microbiota is crucial for a balanced healthy immune homeostasis.

## Pro-And Anti-Inflammatory Dietary Nutrients and Their Role in Intestinal and Systemic Inflammation

While many of gut microbes perform key functions for the host, the host immune system must effectively monitor the microbial community so that interdependency of their relationship is maintained. In order to maintain host homeostasis, the immune system remains tolerant to the diverse microbial community and keep microbes anatomically in check in the gut, while it concurrently maintains the capacity to respond appropriately to microbial attempts to break the intestinal barrier and attack the host (41). This homeostatic state is maintained through pro and anti-inflammatory responses. There is a growing body of evidence that microbiota can impact the stability of pro-inflammatory and anti-inflammatory responses in the gut. Microbial competition for nutrients performs a vital role in controlling this balance (58) and food nutrients with inflammatory potentials can lead to chronic inflammation at intestinal and systemic levels (12).

Western diet is known to induce low-grade intestinal inflammation and is associated with a growing number of diseases (59). Diet quantity, content and timing play a major role in shaping gut microbial composition and function (12). Ingredients in processed food have been reported to selectively promote mucolytic bacterial strains. Chassaing et al. (60) used emulsifiers, detergent-like molecules that are a ubiquitous component of processed foods and that can increase bacterial translocation across epithelia *in vitro*, to study effects on mice gut integrity, microbiota, and immunity. They observed increased levels of several mucolytic operational taxonomic units (OTUs) including *R. gnavus*, and mucosa-associated inflammation-promoting Proteobacteria accompanied with low-grade inflammation and metabolic syndrome in wild type hosts and robust colitis in mice predisposed to this disorder. Similarly, non-caloric artificial sweeteners (NAS), among the most widely used food additives worldwide, have been reported to drive the development of glucose intolerance through induction of compositional and functional alterations in intestinal microbiota (61). Recently, animal food has been associated with lower Bifidobacterium abundance while similar microbial configuration was observed in IBD and IBS (12). A lower population of healthy bacteria (Bifidobacterium and Lactobacilli) have been observed in Western diet compared with Mediterranean diet (3). As discussed here and in section “Inflammation,” these selective alterations in microbiota composition can lead to dysbiosis and shift in metabolites, which negatively impact the gut integrity, and thus facilitate chronic inflammation (2, 10).

A nutritious diet is composed of seven basic components: carbohydrate (excluding fiber), fiber, protein, fat, vitamins, minerals, and water. A balanced diet means eating the correct and right portion of different nutrients on a daily basis. Excess amounts or insufficient amounts of dietary components will affect gut microbiota and consequently affect immune functions. Here, we review several dietary components or foods as examples for their pro- and anti-inflammatory capabilities in order to shed light on their relationship with gut microbiota and the immune system.

### Carbohydrates

Simple carbohydrates (sugars) and refined grains in Western diets and ultra-processed foods and drinks are negatively linked with gut microbiota and the immune system. Added sugars include all sugars that are mainly present in ultra-processed foods and drinks or present naturally in honey, syrups, and fruit juices (62). The harmful effects of sugar-sweetened drinks have been reported for the gut microbiota and their consumption led to increase in the Firmicutes/Bacteroidetes ratio and reduce the proportion of beneficial butyrate-producers such as Lachnobacterium (63). In addition, added sugars elicit increased gut permeability and endotoxemia and consequently induce systemic inflammation and complications (64). Similarly, excessive consumption of fructose in the form of refined sugars is associated with a systemic pro-inflammatory status, cortisol hyperactivation, insulin resistance and increased visceral adiposity (65). Fructokinase C is the key enzyme in the liver to metabolize fructose and it is also adequately expressed in the small intestine. However, fructose metabolism in the intestine leads to disruption of the tight junctions and this effect is not detected in fructokinase knockout mice (66). Further, over-consumption of fructose leads to increase in infiltration of macrophages into adipocytes through monocyte chemoattractant protein-1 and intercellular adhesion molecule-1 (65). Increased number of macrophages into the adipocytes lead to release of pro-inflammatory TNFα leading to further inflammation (67).

Refined grains are grain products consisting of grains or grain flours that have been significantly modified from their natural composition. During process of making refined-grain flour, a higher percentage of these nutrients, ferulic acid (93%); selenium (92%); antioxidant activity (89%); phenolic compounds and magnesium (83%); flavonoids, zinc, and vitamin E (79%); zeaxanthin (78%); fiber (58%); and lutein (51%), are lost (68, 69). Refined grain intake is widely assumed to be associated with adverse health outcomes, including increased risk for cardiovascular disease (CVD), T2DM, and obesity. The 2015 Dietary Guidelines Advisory Committee recommended that to improve dietary quality, the US population should replace most refined grains with whole grains (70). While comparing refined grains with whole grains, the stool weight, stool frequency, and SCFA producer Lachnospira were observed lower while pro-inflammatory Enterobacteriaceae was observed higher in groups fed with refined grains (71). A high-sugar, low-fiber diet for only seven days can worsen Small intestinal bacterial overgrowth (SIBO) and gastrointestinal symptoms (72). On the other hand, whole grains are rich in vitamins, minerals, antioxidants, and other nutrients. Whole grain consumption has been associated with decreased risk of several lifestyle-related conditions including T2DM, CVD, and body weight (73–75). It has been assessed that replacing energy intake from saturated fats with whole grains is associated with lower risk of coronary heart disease while substituting saturated fats with carbohydrates from refine grains is associated with an increased risk of coronary heart disease (76). The germ of whole grains contains a polyamine, named spermidine, which is known to inhibit histone acetyltransferases that results in resistance to oxidative stress and significantly decrease in subclinical inflammation and cell necrosis (77). Further, Roager et al. (73) reported decrease in energy intake, body weight and the low-grade systemic inflammation markers, C-reactive proteins (CRP) and IL-6, in response to whole grain diet in comparison with a refined grain diet, without altering composition of gut microbiome in 8-week diet study, reflecting a direct immune modulating role of whole grains.

### Dietary Fats

High-fat diets are one of the key features of unhealthy eating. In the last few decades, the consumption of fatty acids has increased. In particular, the consumption of saturated fat, trans fatty acids and omega 6 polyunsaturated fatty acids (PUFA) are increasing, while intake of omega-3 PUFA intake is decreasing in developed countries (78). The high-fat diets intake has been associated as a likely cause of gut dysbiosis (79) and linked with gut dysfunction (80), affecting gut microbiota, promotion of intestinal permeability and subsequent inflammation (81). The low-grade inflammation established due to endotoxemia could be linked with development of non-communicable diseases (82). In mice, the high fat diet increased the Firmicutes to Bacteroidetes ratios, promoted pro-inflammatory cytokines such as IFN, TNF, IL-1, and IL-6 (83) and showed endotoxemia (84).

The variants of PUFAs have different metabolites and distinct inflammatory consequences. The omega-6 fatty acids from soybean oil are considered pro-inflammatory while omega-3 fatty acids from fish oil are considered anti-inflammatory (85). The high-fat diets rich in omega-6 polyunsaturated fatty acids (n-6 PUFAs) deplete SCFA-producers in the gut and increase inflammatory CRP levels in humans (86). In opposite, the n-3 PUFA show its anti-inflammatory effects by inhibiting production of pro-inflammatory cytokines (IL-1β, TNF-α, and IL-6), chemokines (IL-8), mediators (leukotrienes), and reactive oxygen and nitrogen species (87–89). The n-3 PUFA block NF-kB signaling, a transcription factor that control most of the pro-inflammatory mediators, possibly through interfering with the TLR4 and its receptor protein MyD88 and activating anti-inflammatory transcription factor PPARγ (90, 91). The n-3 PUFAs (LC n-3 PUFAs), eicosapentaenoic acid and docosahexaenoic acid, are acted as precursors for lipid mediators, resolvins and protectins, that actively alleviate and/or resolve inflammation (92). Further, n-3 PUFA have been observed to decrease pro-inflammatory T cell subsets (Th1, Th17) and activate anti-inflammatory T cell subsets (Th2, Treg) (93). Due to these anti-inflammatory effects, protective role of n-3 PUFA have been reported in different chronic inflammatory conditions like CD, UC, and rheumatoid arthritis (94, 95). The ratio of omega-6 to omega 3 fatty acids reaches to 15:1 in western style diets due to refined oils (78) and increase metabolic endotoxemia through interactions with gut microbiota (96). The modified omega 6 to omega 3 ratio increases abundance of Enterobacteriaceae, segmented filamentous bacteria and Clostridia spp. and promotes a pro-inflammatory condition that could be lessened by increasing omega 3 PUFA levels (97).

Supplementation of extra-virgin olive oil in clinical trials showed a significant reduction in circulating oxidized low-density lipoprotein (LDL) and inflammatory markers (98). Extra virgin olive oil contains a phytochemical, olechantal, that shows an ibuprofen-like COX-inhibitory activity (99). In contrast, refined olive oils decrease the abundance of favorable bacterial families such as Erysipelotrichaceae and Sutterellaceae and increase in non-beneficial bacteria with negative consequences for the immune system such as Desulfovibrionaceae, Spiroplasmataceae, and Helicobacteraceae (100). Similarly, refined palm oil has been reported to have negative effects on microbiota and intestinal integrity and promote release of pro-inflammatory cytokines (101). Palmitic acid has been reported to induces IL-1β-mediated inflammation by TLR4 signaling, which consequently lead to activation of the NLRP3 inflammasome (102). Further, intake of trans fatty acid has been associated with elevated inflammatory markers (CRP, IL-6, and tumor necrosis factor receptor 2) and an increased risk of developing cardiovascular problems (103).

### Proteins and Amino Acids

Proteins and their constituents in diets affect host gut microbiota (104) and immune system (105). These proteins in intestine act as substrates for digestive enzymes and microbial fermentation and affect the diversity and composition of gut microbiota. For instance, the hen egg white promotes abundance of Akkermansia in rats while duck egg white facilitates abundance of Proteobacteria and Peptostreptococcaceae (106). It has been observed that a diet high in protein reduces abundance of propionate and butyrate producing bacteria (*Ruminococcus*, *Akkermansia*, and *F. prausnitzii*) and promotes conducive conditions for pathogenic bacteria such as *Escherichia/Shigella*, *Enterococcus*, and *Streptococcus* (107, 108). In addition, a low protein diet may lead to higher abundance of Desulfovibrionaceae that is positively correlated with inflammation and lead to intestinal infections (109), reflecting an optimum protein intake is required for gut health. Different human studies have reported a link between a high dietary protein intake and increased risk inflammatory conditions like IBD and its relapse (110–112). A study using the murine colitis model has showed that a diet high in red meat exacerbates the disease index in comparison with a casein-based protein diet (113). Processed and red meat may be associated with certain pathological conditions, particularly colorectal cancer, and many mechanisms, including involvement of gut microbiota, have been proposed (114). Among them, red and processed meats contain elevated levels of L-carnitine that is considered the precursor for trimethylamine (TMA) produced by the gut microbiota (115). The TMA in the liver is transformed into trimethylamine N-oxide (TMAO) that is linked with inflammatory pathways and cardiovascular disease risk (116). Higher TMAO levels are positively associated with monocyte activation and induction of NLRP3 inflammasome, which subsequently trigger inflammatory immune responses (117). Further, red and processed meats contain elevated levels of heme iron that has been correlated with an hyperproliferation of enterocytes and the change in the intestinal barrier (118). Alterations in the abundance of certain microorganisms such as *Fusobacterium nucleatum*, *S. bovis*/*gallolyticus*, *E. coli*, and Bacteroides fragilis have been reported due to excessive red meat consumption (119). Recently, processed foods and animal-derived foods have been associated with higher abundances of Firmicutes, Ruminococcus species of the Blautia genus and endotoxin synthesis pathways (12).

Growing literature has showed that amino acids can influence the composition and functionality of gut microbiota (120, 121). These amino acids are metabolized through gut microbiota into different metabolites including polyamine, SCFA, phenol and indole that are involved in various physiological functions and are related to host health and diseases (121). It has been observed in mice fed with methionine-restricted diets that abundance of SCFA-producing bacteria (*Bifidobacterium*, *Lactobacillus*, *Bacteroides*, *Roseburia*, *Coprococcus*, and *Ruminococcus*) and inflammation-inhibiting bacteria (*Oscillospira* and *Corynebacterium*) has been increased while abundance of inflammation-causing bacteria (*Desulfovibrio*) has been decreased, thus conferring health benefits to the host (122). Specific amino acids have also been discovered to modulate the immune system. Arginine supplementation affects T lymphocyte response and increase CD4 + T helper (Th) cells population in postoperative cancer patients (123). Dietary arginine and glutamine have significantly decreased colonic IL-17 and TNF-α cytokines in a dextran sulfate sodium-induced colitis mice model (124). In another study, IBD patients with active disease have shown higher levels of tryptophan metabolites, especially quinolinic acid, suggesting a probable role of tryptophan in IBD pathogenesis (125). These studies demonstrate that proteins and amino acids have impact on gut microbiota and immune system and can modulate gut homeostasis. An optimum level of dietary proteins and amino acids is required for good gut health while higher and lower dietary levels may favor pro-inflammatory gut microbiota and inflammation.

### Salts

The excess intake of salt has been associated with a heightened risk of developing high blood pressure (126), cancer (127), and chronic inflammation (128). Furthermore, there is growing evidence that excess salt consumption impacts the immune system (129). The high salt diets have been associated with activation of inflammatory and inhibition of anti-inflammatory responses. Wei et al. (130) showed that mice on a high-salt diet showed higher number of pro-inflammatory IL-17A producing cells in the intestinal lamina propria in comparison with mice on a normal salt diet. Similarly, human intestinal mononuclear cells increased production of IL-17A, IL-23R, TNFα and RORγt when exposed to high concentrations of salt (131). Moreover, ingestion of high amounts of salt also significantly suppressed Treg cells and their anti-inflammatory function by repressing IL-10 secretion in mice and human (130, 132, 133).

High concentrations of salts could also negatively affect the gut microbiota. High-salt diets decrease the abundance of *Oscillibacter*, *Pseudoflavonifractor*, *Clostridium XIVa*, *Johnsonella*, and *Rothia*, while the abundance of other species is increased, including *Parasutterella* spp. *Erwinia* genus, Christensenellaceae, Corynebacteriaceae, Lachnospiraceae, and *Ruminococcus* (134). Wilck et al. (135) have reported the direct effects of high salt concentrations on the gut microbiota-Th17 axis, especially reducing the abundance of Lactobacillus species, which could be the reason of altered production of SCFA and worsening of colitis in high-salt diets (136).

### Vitamin D

The extra-skeletal effects of vitamin D are well documented for the immune system. Many immune cells express vitamin D receptors (VDR) and both systemic and locally generated vitamin D in its active form can modulate innate and adaptive immune responses (137). Vitamin D hinders T cell effector functions (138, 139), especially production of cytokines such as IL-2 and IFN-γ. Further, vitamin D favors differentiation of T helper cells toward regulatory Th2 and development of Treg cells and inhibits polarization toward pro-inflammatory Th1 and Th17 cells (139, 140).

### Zinc

The transition metal zinc is an important micronutrient and is required to control different key biological processes including growth, repair, metabolism, cell integrity, and functionality (141). Besides boosting defense-related immune functions, the importance of zinc in maintaining immune tolerance is well-established. Zinc has been shown to play a role in development of Treg cells (142, 143), and inhibiting pro-inflammatory Th17 and Th9 cell differentiation (144, 145). It has been observed that zinc promotes bone marrow-derived dendritic cells to develop into tolerogenic phenotype by inhibiting MHC-II expression and tilt Treg-Th17 balance in favor of Treg cells (146).

Deficiencies and excess in micronutrient can shift the intestinal and systemic homeostasis toward pro-inflammatory arm and play an important role in local and systemic inflammation through changes in gut microbiota. Therefore, targeting gut microbiota, as considered by some investigators as a single organ (147), through different interventions to reprogram its structure and function for alleviation of inflammation, provides new potent opportunities. Intervention through whole food with anti-inflammatory abilities holds preventive and therapeutic potentials against inflammation but limited data in healthy and disease conditions and difficulty to intervene through dietary change are the limitations. However, the knowledge on pro-inflammatory and anti-inflammatory capacities of single compounds is increasing through functional experiments (12). But these single compound trials do not acknowledge the interactions of nutrients within their food matrix, which may explain the contradictory and less efficacious outcomes of these trials (148). Here, we will discuss the potential role of other microbiome-based interventions like probiotics, prebiotics and synbiotics in the gut and systemic inflammation.

## Potential Role of Microbiome-Targeted Interventions in Intestinal and Systemic Inflammation

### Probiotics

Probiotics are defined as “live microorganisms that, when administered in adequate amounts, confer a health benefit on the host” (149). Probiotics are increasingly incorporated into a wide range of foods, beverages, and topical products (16). The probiotic industry is growing annually at estimated rate of 7% (150). The health benefits of probiotics have been documented in humans (151), livestock (152), and poultry (153–155). Probiotic preparations are mostly based on Bifidobacterium and Lactobacillus spp. Clinical evidence supports the use of probiotics for treating intestinal inflammatory conditions (156–158).

**FIGURE 3:**
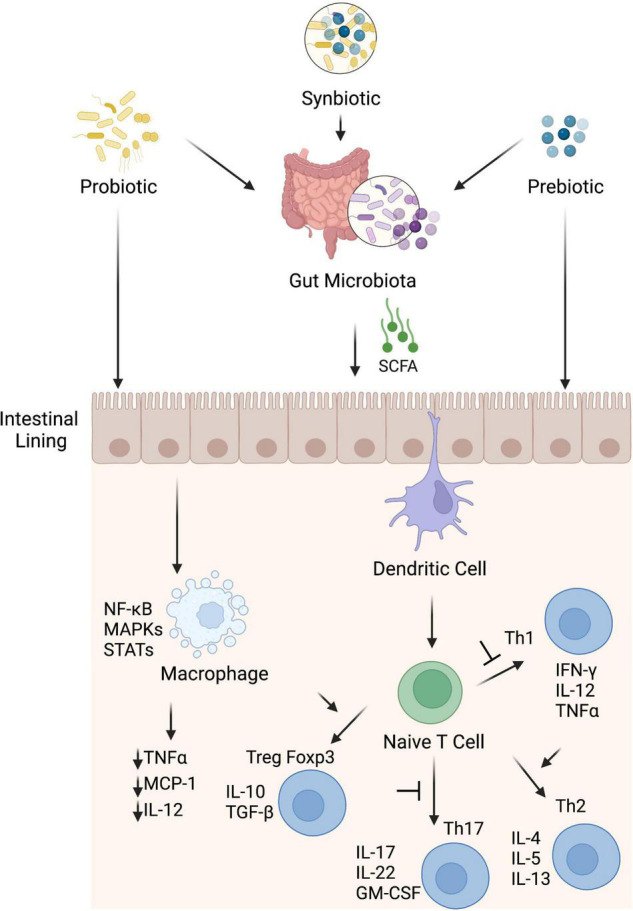
Potential anti-inflammatory mechanisms induced by probiotics, prebiotics and synbiotics. Probiotic, prebiotic and synbiotic can stimulate anti-inflammatory components of the immune system directly or indirectly through modulation of gut microbiota. These interventions interact with sentinel cells such as dendritic cells and macrophages through membrane receptors e.g., PRRs and suppress inflammation. Dendritic cells induce anti-inflammatory state through blocking transformation of naïve T cells into Th1 and Th17 cells and promote proliferation of Treg cells that consequently with help of their cytokines like IL-10 and TGF-β skew immune homeostasis toward anti-inflammatory state. Macrophages, once activated, produce different types of pro-inflammatory cytokines to promote inflammation but microbiota derived SCFA, especially butyrate, hinder different pathways like MAPK, STAT and NF-kB and suppress pro-inflammatory cytokines (monocyte chemotactic protein-1, TNF-α, and IL-12) from macrophages to suppress inflammation.

The intestinal homeostasis is strongly affected by the interactions between the mucosal immunity and the microbiota that coexist in a mutually beneficial relationship (159). The positive effects of probiotics on gut health have been ascribed to different mechanisms including their direct and indirect immunomodulation (160). Such immunomodulation varies from immune stimulation of immune responses by *L. rhamnosus* GG, *Lactobacillus acidophilus*, and *Lactobacillus casei* (161, 162) to tolerance brought by *Lactobacillus plantarum* (163). Probiotics can interact directly with the host through their surface cell wall effector molecules like peptidoglycan and lipoteichoic acid and specific proteins (164). These interactions affect intestinal epithelial, enteroendocrine, immune and vagal afferent nerves and reflect local intestinal effects such as development of intestinal barrier integrity and inflammation (as discussed in section “Inflammation”) and systemic effects via host immune, endocrine, and nervous system mediators. Probiotics can also affect host indirectly through interactions with intestinal microbiota and their secreted metabolites. These include cross feeding of microbiota through metabolites, changes in the intestinal microenvironment like by reducing pH, occupation of binding sites and nutrients competition, and inhibition of growth through the secretion of strain-specific antibacterial compounds (165–167). The probiotic-mediated direct immunomodulatory effects may be prominent in less dense commensal areas like small intestine (168) in comparison with likely indirect effects through endogenous microbiota in densely populated areas like colon (169). Certain probiotics induce anti-inflammatory cytokines that reduce the induction of inflammation (170), while others alter the diversity of microbiota that promotes anti-inflammatory state in the gut (Figure 3) (171, 172). Lactobacillus and Bifidobacterium species have been shown to stimulate Treg cells and upregulate anti-inflammatory cytokines like IL-10 and transforming growth factor-β (TGF-β) (173, 174). These studies reflect that probiotics have potential to modulate gut immune responses directly or indirectly and thus affect the gut homeostasis.

Probiotics have been used to alleviate food borne intestinal and systemic inflammation. A probiotic strain of *Lactobacillus reuteri* has been used for restoration of the gut barrier defect in dietary- and genetic- induced metabolic impairment through production of aryl hydrocarbon receptor ligands that repaired leaky intestinal mucosa and improved consequent inflammatory complications (175). Recently, mice fed a high-fat diet (HFD) with live *Dysosmobacter welbionis* probiotic strain has shown reduction in pro-inflammatory cytokine, TNFα, and several macrophages infiltration markers such as CD68, CD163, and CD11b and decrease in body weight, fat mass gain and insulin resistance (176). Similarly, Wang et al. (177) reported improvement in ileal tight junction protein expression and reduction in endotoxemia, weight gain, hyperglycemia and hepatic steatosis in HFD-fed mice in response to *Parabacteroides distasonis*. The probiotic strain of *Akkermansia muciniphila* has been suggested to strengthen mucus thickness and tight junction expression in intestine for improvement of gut leakiness in mouse model for alcoholic liver disease (178). In the same manner, *Bifidobacterium longum* supplementation protected against fiber-depleted diet induced colonic mucus deterioration and improved gut health (179).

It seems that surface as well as secreted components from lactobacilli play an important role in induction of anti-inflammatory state. A protease (lactocepin) secreted by *Lactobacillus* species degrades IP-10, a lymphocyte-recruiting chemokine, in the intestine that lowers cecal inflammation in a colitis model (180). Another protein, p40, secreted by *Lactobacillus* species has been shown to decrease inflammation related cytokines and protects against colitis in mice (181). Further, the *L. plantarum* (182) and *Lactobacillus rhamnosus* GG probiotic strains with mutations that altered the D-alanine display on lipoteichoic acid or lipoteichoic acid-deficient *L. acidophilus* probiotic strain (183) showed anti-inflammatory effects and prevented or reduced colitis symptoms in mice. In the same manner, *Bifidobacterium breve* has been reported to reduce pro-inflammatory cytokines (184) and LPS-induced epithelial cell shedding (185) while *F. prausnitzii* and spore-forming Clostridium clusters IV and XIV with strong anti-inflammatory effects have been proposed as probiotic supplement to induce tolerance and lessening inflammatory symptoms (46, 186, 187). Probiotics have shown promising results in clinical trials related with several inflammatory conditions (Table 1). For example, Bjarnason et al. (188) reported decreases in fecal calprotectin in IBD patients while using a cocktail of probiotic strains. Similarly, Andresen et al. (189) reported improvement in symptoms of IBS patients in response to heat-inactivated *Bifidobacterium bifidum* probiotic strain.

**TABLE 1:** Important clinical trials showing effects of prebiotics, probiotics and synbiotics on inflammatory conditions.

Study (Ref)	Intervention	Intervention type	Condition	Duration	Effect of intervention
Bjarnason et al. (188)	Probiotic	*Lactobacillus rhamnosus, Lactobacillus plantarum, Lactobacillus acidophilus, and Enterococcus faecium*	IBD	4 weeks	Decrease in fecal calprotectin (FCAL)
Andresen et al. (189).	Probiotic	Heat-inactivated *Bifidobacterium bifidum*	IBS	8 weeks	IBS symptoms improved
Ligaarden et al. (192).	Probiotic	*Lactobacillus plantarum*	IBS	6 weeks	IBS symptoms deteriorated
Besselink et al. (193).	Probiotic	*Lactobacillus acidophilus, Lactobacillus casei, Lactobacillus salivarius, Lactococcus lactis, Bifi dobacterium bifi dum, and Bifi do-bacterium lactis*	acute pancreatitis	28 days	Increased risk of mortality
Dehghan et al. (229).	Prebiotic	Oligo fructose enriched inulin	T2DM	8 weeks	Decrease in IL-12 and IFN-γ
Dehghan et al. (230).	Prebiotic	Oligo fructose enriched inulin	T2DM	8 weeks	Decrease in TNF-α and IL-6
van den Berg et al. (231).	Prebiotic	Fructo-oligosaccharide/Galacto-oligosaccahride	Preterm infants	27 days	Decrease in TNF-α, IFN-γ, and IL-1β
Abbas et al. (251).	Synbiotic	*Saccharomyces bourardii* + psyllium	IBS	12 weeks	Decrease in IL-8 and TNF-α
Akram et al. (252)	Synbiotic	1 synbiotic capsule	T2DM	8 weeks	Decrease in CRP, IL-6, TNF-α
Eslamparast et al. (253)	Synbiotic	*L. casei, L. rhamnosus, S. thermophilus, B. breve, L. acidophilus, B. longum, L. bulgaricus* + Fructo-oligosaccharide	NAFLD	28 days	Decrease in CRP and TNF-α

Despite many benefits of probiotics to the gut health, some probiotics show opposite or no results in the host. *L. plantarum* v299 strain has been reported as a potent pro-inflammatory probiotic strain, which showed crypt destruction and the recruitment of inflammatory cells in the intestine (190). Two *Lactobacillus* strains exacerbated experimental colitis when administered in mice (191). Similarly, administration of *L. plantarum* MF1298 strain showed detrimental effects in subjects of IBS (192). It was observed in patients with acute pancreatitis that their mortality increased after administration of a combination of three probiotics (193). It seems that effects of probiotics are dependent on probiotic species and the strains, as some strains have anti-inflammatory effects while others are pro-inflammatory. Further, same probiotic strains can have profoundly different effects on the host, differing in survival in gut environment, adherence to epithelial lining, and pathogen inhibition (194, 195), probably due to the difference of existing gut microbiome in the host. The baseline microbiota shows a degree of colonization resistance to probiotics, which can influence the duration of residence, penetration into the mucosal microbiome and potential impact of biological activity (196). Different studies have demonstrated individual specific variability in probiotic colonization, either persistently or transiently during probiotic supplementation (197–199). Zmora et al. (198) reported that host immune factors and composition of microbiome as determinants of mucosal colonization of administered probiotics. These studies reflect that outcome of probiotic use may be determined by different factors including strain and species characteristics, enterotype of microbiome (200) and host responses.

### Prebiotics

Prebiotics are defined as “a selectively fermented ingredient that results in specific changes in the composition and/or activity of the gastrointestinal microbiota, thus conferring benefit(s) upon host health.” Potential mechanisms of action of prebiotics have been proposed (201). The gut microbiota can ferment prebiotics into SCFA, mainly acetate, propionate and butyrate. SCFA lower the luminal pH, provide energy sources for epithelial cells and have profound effects on inflammation modulators (Figure 3 and section “Inflammation”) and metabolic regulations. Oligosaccharides and monosaccharides can also reduce pathogen colonization by blocking the receptor sites used by pathogens for attachment to the epithelial cell surface (201).

A large number of microorganisms lives in GI tract, and it has been reported that there are 10^10^–10^12^ microorganisms in human colon (202). These microorganisms are sustained by diet components as well as endogenous energy sources like mucin. In particular, non-digestible components in diets have influence on composition and function of microorganisms. These non-digestible dietary substances are used in fermentation by beneficial microbiota to obtain energy for their survival (203, 204).

A good prebiotic product should show characteristics of resistance to acidic pH and enzymes in the gut, resistance to absorption in the gastrointestinal tract, be fermentable by intestinal microbiota. They will selectively stimulate the growth and/or activity of the gut bacteria and improve host’s health (205, 206). Prebiotics mainly include fructans (inulin and fructo-oligosaccharide), galacto-oligosaccharides, starch and glucose-derived oligosaccharides (207, 208). Prebiotics can selectively influence the gut microbiota. Prebiotics confer their beneficial effects on host through direct interactions with gut mucosal tissues or indirectly through gut microbiota Figure 3. Previously, population of Lactobacilli and Bifidobacteria were the main target for prebiotics to have beneficial effects (209) but now the list of targets has been expanded to other health-promoting genera such as *Roseburia* spp., *Eubacterium* spp., *Akkermansia* spp., *Christensensella* spp., *Propionibacterium* spp., *Faecalibacterium* spp., and *Anaerostipes* spp. (210, 211). In addition to targeted groups within the microbiota which can utilize prebiotics directly, other bacterial groups within the microbiota may also benefit through process of cross feeding (212–214). For example, *Ruminococcus bromii* can degrade resistant starches, and several other species can utilize the fermentation products of this reaction (215). Consequently, changes in composition and metabolites of microbiota lead to impact on host epithelial, immune, nervous, and endocrine systems and promote health benefits (212). One of main by-products of bacterial prebiotic metabolism are the SCFA, acetate, propionate and butyrate, that are well known to impact host systems and mediate many prebiotic effects (210). SCFA are small molecules and can pass through gut epithelial lining and enter blood circulation. Hence, they can affect gastrointestinal track as well as distant site organs and systems (216). There is evidence that prebiotics can directly regulate host in microbe-independent mechanism. Prebiotic molecules interact directly with host receptors, affecting epithelial and immune cells signaling pathways and regulate barrier function and inflammation (217). In this regard, the immune effector cells become hyporesponsive to activate NF-κB and mitogen-activated protein kinase (MAPK) after exposure to oligosaccharides and consequently regulate inflammation via direct modulation of kinome instead of altering gut microbiota (1). Human milk oligosaccharides (HMOs) may also interact directly with host gut epithelial or immune cells for its immunomodulatory effects (218).

Prebiotics have been shown to reduce enteric and systemic inflammations. The abundance of Bifidobacteria and *F. prausnitzii* along with Bacteroides to Firmicutes ratio is decreased in intestinal inflammation (219, 220). Prebiotics like galacto-oligosaccharides (GOS) and fructo-oligosaccharides (FOS) have been observed to improve microbial profiles by elevating bifidobacteria and decreasing *E. coli* (151, 221). The population of Bifidobacteria was elevated in feces and the anti-inflammatory function of intestinal dendritic cells was improved when human subjects were supplemented with 15 g/day FOS for 3 weeks (222). Supplementation of oligofructose in obese mice improved gut barrier integrity and systemic and hepatic inflammation, possibly through a GLP2-dependent mechanism (223). Supplementation of FOS normalized insulin resistance, leptin levels, dyslipidemia and osteoarthritis in diet induced obese rats (224). Inulin alone or in combination with probiotic or butyrate are beneficial in conditions like UC (225, 226), obesity (227), and T2DM (228). Clinical studies on effects of using prebiotics for alleviation of inflammation in inflammatory conditions have shown favorable results (Table 1). The use of fructo-oligosaccharide, galacto-oligosaccahride and inulin reduced inflammatory markers in T2DM patients (229, 230) and Preterm infants (231).

Prebiotics may fail to provide the expected benefits or even show opposite outcomes. A double-blind cross-over study showed that the supplementation of oligofructose at the dose of 6 g/day for 4 weeks to IBS patients showed no improvement in symptoms (232). In another randomized, double-blind, placebo-controlled study, 20 g/day FOS supplementation failed to improve symptoms in IBS patients (233). Similarly, supplementations of FOS at rate of 15g/day (234) and oligofructose-enriched inulin at rate of 20 g/day (235) in patients with CD did not show significant clinical benefits. Further, in one study the IBS symptom scores even got worsen when supplemented with FOS prebiotic (236).

These differences in outcomes of prebiotics could be attributed to different enterotypes of microbiota in different individuals (200). Specific prebiotics may be more suitable to present a health benefit when they are given to individuals with appropriate baseline microbial configuration. Differences in the gut microbiome have also been associated to differential clinical response to prebiotics in terms of stool consistency (237) in healthy adults and hepatic lipid metabolism in hepatic steatosis patients (238). In another study the production of SCFA was compared across FOS, sorghum and arabinoxylan and it was observed that volunteers with fiber-utilizing Prevotella dominated microbiota gave equally high response to each fiber than volunteers with Bacteroides-dominated microbiota (239). Recent dietary intervention studies with healthy European subjects pointed to a more beneficial role of a high-fiber diet for individuals with a Prevotella enterotype than individuals with the Bacteroides enterotype (240). So, as the enterotypes seem to differ in their ability to degrade substrates (241), it can be speculated that metabolic responses of the various enterotypes would be different and thus affect health outcomes.

### Synbiotics

Synbiotics are products that contain both probiotics and prebiotics, and this mixture is supposed to be more efficient compared to individual components alone, in terms of gut health and function (212). The components of the synbiotics may be complementary or synergistic to each other. These may be complementary that mechanisms of action of each component can be independent of each other and both components have their own demonstrated health benefits. These may be synergistic in the sense that synbiotics include a fermentable substrate (prebiotic) for the co-administered live microbe (probiotic), where the substrate and the microbe may or may not be able to elicit a health benefit independent of the other, but they have a proven health benefit in combination (242). Supplementation with synbiotics is supposed to show a better effect on intestinal and systemic inflammation than prebiotics or probiotics alone because of their superior ability to increase SCFA producing bacteria and providing substrates for their fermentation (243). SCFA promotes mucosal barrier stabilization, T reg cell induction, anti-inflammatory cytokines secretion and inhibition of inflammatory factors (244, 245) (Figure 3).

Synbiotics have been observed in lessening the gut and systemic inflammation and in some instances, they showed superior effects over prebiotics and probiotics. For example, Vu et al. (246) compared effects of prebiotics, probiotics and synbiotics on obesity-associated colitis and hepatic manifestation and observed that prebiotic and probiotic alone did not modulate inflammatory cell infiltration and inflammatory markers (i.e., IL-6 and TNF-α). Similarly, In IBD patients, the preliminary evidence suggested that synbiotics were more effective than probiotics or prebiotics alone (247). Yao et al. (248) reported a role of synbiotics in alleviation of high fat diet-induced hepatic steatosis, release of TNF-α and decrease in the progression of cirrhosis. They observed a transcriptional decrease in inflammatory factors such as lipopolysaccharides, TLR-4 and NF-κB and improvement in gut integrity and function. Li et al. (249) reported that synbiotics mediated improvement in the body weight, epididymal fat index, blood lipid level, and liver function indexes of mice with diet induced hyperlipidemia. The synergistic effects were also observed in the mRNA expressions of ZO-1, occludin, and claudin-1 in the small intestine, strength of the intestinal barrier, and composition of the intestinal microbiota. Beneficial effects of synbiotics were also observed in NAFLD and was proposed as a probable management strategy for patients with NAFLD (250). Clinical data also supported the use of synbiotics (Table 1), where synbiotics significantly reduced inflammatory markers in IBS (251), T2DM (252) and NAFLD (253) patients.

The synbiotics may fail to show beneficial effects or worsen the inflammatory conditions. In a meta-analysis conducted by McLoughlin et al. (254), 10 out of 26 studies in humans indicated no change in inflammatory biomarkers while 2 other studies showed an increase in inflammatory parameters. Formulation of synbiotic ingredients and dosage may be likely factors to explain conflicting outcomes of these studies in terms of anti-inflammatory effects of synbiotics.

### Effects of Probiotics, Prebiotics and Synbiotics in Undernutrition/poor Diet Conditions

One aspect of poor nutrition is undernutrition that leads to stunting (low height for age), wasting (low weight for height), underweight (low weight for age) and micronutrient deficiencies or insufficiencies (a lack of important vitamins and minerals) (255). Probiotics, prebiotics and synbiotics have been documented to ameliorate the effects of poor diets in terms of undernutrition. However, dietary interventions alone may not be insufficient to comprehensively reduce the burden of undernutrition. Undernourished children have been observed with immature, less diverse, and dysbiotic gut microbiota which might be modified using gut microbiota–targeted nutritional interventions (256). In a recent systemic review encompassing studies in low- and middle-income countries, probiotics and synbiotics were reported to have potential advantages to improve the growth of undernourished children (257). Similarly, Onubi et al. (258) concluded in their systemic review that probiotics have the potential to improve child growth in developing countries and in under-nourished children. Castro-Mejía et al. (259) used probiotic strains (*L. rhamnosus* GG and *Bifidobacterium animalis* subsp. lactis BB-12) for restitution of gut microbiota in Ugandan children with severe acute malnutrition and reported an advantageous increase in microbial observed species and reduction in the cumulative incidence of diarrhea during the outpatient phase. In this context, the effect of prebiotics in undernourished is not supported by strong experimental evidence. Heuven et al. (257) found no effect of prebiotics in low- and middle-Income countries in their systemic review while Mugambi et al. (260) observed increase in the abundance of Bifidobacteria and weight gain of full-term healthy infants in response to prebiotics. The response of healthy children in comparison with undernourished children might be different to prebiotics as undernourished children have immature, less diverse, and dysbiotic gut microbiota (256). In this regard synbiotics showed improvement in gut microbiota of young children. Chua et al. (261) used a synbiotic in infants with a compromised microbiota at birth and observed that the synbiotic caused earlier colonization of the gut microbiota by Bifidobacteria. Similarly, Kosuwon et al. (262) reported to use synbiotic consisting of scGOS/lcFOS and *Bifidobacterium breve* M-16V, for increasing abundance of fecal Bifidobacterium in healthy young children. Another advantage of using this approach is its practicality, as most prebiotics, probiotics and synbiotics are inexpensive, flavored, easy to be administered and available over the counter. Despite the beneficial effects of these remedies, major disadvantages/limitations of using these approaches are the possibility of development of resistance as the bacterial strains used might have the capacity for horizontal gene transfer of antibiotic resistance genes and inconsistent results. However, majority of the studies have showed potential of probiotics, prebiotics and synbiotics in undernourished children. Nevertheless, more detailed studies in different socioeconomic conditions are required to support claims for beneficial effects of these interventions.

## Strengths and Limitations of the Study

This review has examined recent research on food borne inflammation, involvement of gut microbiota and immunity in such inflammatory processes, and potential roles of gut microbiota targeted interventions like prebiotic, probiotic and synbiotic in amelioration of gut and systemic inflammatory conditions. However, the present review did not encompass the effects of several other emerging microbiome targeted interventions such as parabiotics, fermented foods, phytochemicals, vitamins, and minerals on gut and systemic inflammation.

## Conclusion

**FIGURE 4:**
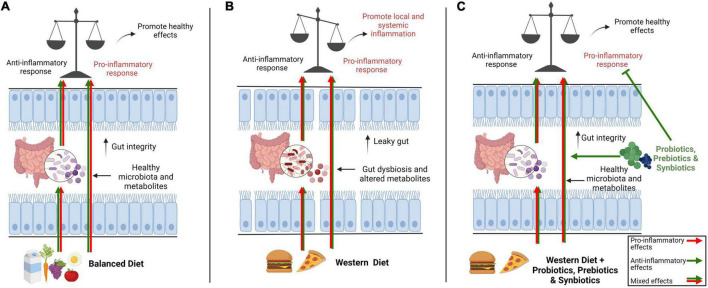
Overview of the impact of a balance diet versus western diet on gut microbiota and immune system and potential roles of interventions such as prebiotics, probiotics and synbiotics on microbiota-immune axis. In general, a balance diet modulates gut immune system directly or indirectly through gut microbiota with a mix of pro- and anti-inflammatory signals so that the immune system stays alert to keep its defenses but also remain tolerant to unnecessary signals to avoid needless inflammation **(A)**. The pro-inflammatory components in western diet including low-quality fats (trans fatty acids and refined oils), refined carbohydrates (sugar and refined grains), unhealthy additives, processed red meat and salts directly modulate the immune system or negatively impact gut microbiota, causing dysbiosis and changes in metabolites leading to a leaky gut. Consequently, the gut immune homeostasis is shifted toward local or systemic chronic inflammation **(B)**. Different interventions like prebiotics, probiotics and synbiotics can potentially amend dysbiosis and gut barrier integrity by promoting healthy gut microbiota and conferring their anti-inflammatory effects on immune system to restore gut immune homeostasis **(C)**.

Inflammation is the response by host tissues to tackle endogenous and exogenous dangers. It varies from low grade subclinical to severe and clinically observable forms with variable durations. Western diets contain various dietary components which cause low-grade inflammation in the gut. The dietary component-induced inflammation may lead to many gut and ex-gut inflammatory diseases such as IBS, CD, obesity, T2DM and cardiovascular problems. Gut microbiota remains in continuous interaction with the host immune system, and changes in microbial configuration and metabolism have direct consequences on immune responses as explained in Figure 4. Foods can be seen as pro- and anti-inflammatory, depending on the immune response induced. Shifting from Western style food to Mediterranean or fermented type of food will confer anti-inflammatory effects on a host but might be a difficult and less sustainable choice. As a supplemental choice, microbiome-based interventions such as usage of probiotics, prebiotics and synbiotics may help in prevention as well as therapy of inflammatory conditions that are associated with gut dysbiosis and immune dysregulation. However, caution must be exercised when using probiotics, prebiotics and synbiotics since there are reports that show inconsistencies and undesired/opposite outcomes. Future investigations to develop next generation of probiotics, prebiotics and synbiotics will help to fill the lacunae left behind by classical probiotic and prebiotic approaches.

## Author Contributions

All authors listed have made a substantial, direct, and intellectual contribution to the work, and approved it for publication.

## Conflict of Interest

The authors declare that the research was conducted in the absence of any commercial or financial relationships that could be construed as a potential conflict of interest.

## Publisher’s Note

All claims expressed in this article are solely those of the authors and do not necessarily represent those of their affiliated organizations, or those of the publisher, the editors and the reviewers. Any product that may be evaluated in this article, or claim that may be made by its manufacturer, is not guaranteed or endorsed by the publisher.
